# What You Cannot Do with Purgatives

**Published:** 1907-11

**Authors:** Edwin Walker

**Affiliations:** Evansville, Ind.


### Journal-Record of Medicine

Successor to Atlanta Medical and Surgical Journal, Established 1855, and Southern Medical Record, Established 1870.

**OWNED BY THE ATLANTA MEDICAL JOURNAL CO.**

**Published Monthly.**

*Official Organ Fulton County Medical Society, State Examining Board, Presbyterian Hospital, Etc.*

VOL. IX. NOVEMBER, 1907. No. 8

BERNARD WOLFF, M. D., EDITOR, 319-320 Prudential Bldg.

GEORGE BROWN, M. D., BUSINESS MANAGER, Business Office 1½-5½ S. Broad St.

E. G. BALLENGER, M. D., ASSOCIATE EDITOR AND ASSISTANT BUSINESS MANAGER.

### ORIGINAL COMMUNICATIONS.

### WHAT YOU CANNOT DO WITH PURGATIVES.*

By EDWIN WALKER, M.D., Evansville, Ind.

In spite of the rigid criticism and enquiry of our age, there is still in medical literature much which is untrue. An incorrect observation or an immature conclusion by some recognized authority is quoted for years, perhaps for generations, without a question. The medical profession is swayed by fads and fancies, as well as the laity, and possibly in no less degree.

Brown-Sequard, like many other brilliant but erratic teachers, led the profession astray for almost half a century by his views on reflex irritation as a cause of disease. The proprietary medicine man has reaped his harvest through the credulity of the doctor. Bare assertions of the venders were enough in most cases, but there has been no difficulty in obtaining the most glowing tributes from reputable physicians to boom their wares when interest waned.

It behooves us, therefore to carefully analyze and sift everything and accept only what rests on scientific demonstration.

*Read before the Mississippi Valley Medical Association, October 8-10 1907, Columbus, O

The use of purgatives has been so general, from the beginning of medical knowledge, one would expect to find distinct and clear exposition of their mode of action, and definite and reliable indications for the use, and with this their contraindication and dangers.

A brief review of the subject in recent works on therapeutics will convince anyone that our knowledge is far from satisfactory, and that but little of value has been added to our knowledge in the past decade. If you seek to learn in what manner they act, you will find that about the only point on which all are agreed is that they are irritants, and when excessively used produce inflammation. The resemblance in action of purgatives and infections has been pointed out (Walsh.) Roos found he could produce purgation by feeding live cultures of the colon bacillus, while dead ones had no such effect.

On the other hand, A. Schmitt, Strasberger, and others have found that constipated stools contain fewer germs, and undergo putrefaction slowly, even in an incubator.

Clinical observation confirms this. Purgatives produce liquefaction of feces, by a fluid which cannot be differentiated from an inflammatory exudate, increased formation of gas and griping. In what does this picture differ from enteritis due to infection? There is most probably increase in germ life in both.

The purgatives most frequently prescribed are calomel, salines and castor oil.

In spite of its general use and repeated experiments on animals, the method and nature of the action of calomel on the human system are in dispute. It is claimed that its action is due to its conversion into the bichloride, and this is denied by the best authorities. Good authorities teach that it increases the flow of bile, while those equally as distinguished tell us that it diminishes it. Whether it acts on the liver cells or not is unknown. That it does powerfully affect the human system and in large doses is a violent poison is admitted by all. It does increase bacterial growth in the intestine, as its excessive use will cause enteritis.

Is this, then, a drug to be given as a routine? Should it not be withheld except when indications are clear? Should not its contraindications be impressed emphatically on every physician?

In regard to the action of salines we are equally uncertain. Their action by osmosis or by diminishing the absorption is hardly tenable. The result of experiments on animals, as well as clinical evidence, seems to discredit this view. On the other hand, the increase of albuminous exudate and gas, with the well-known irritating qualities of strong solutions, render it much more probable that they also act as irritants and increase germ activity in the intestines, and, if used excessively, produce inflammation.

They are doubtless useful in some conditions, although I am not quite sure that much can be accomplished by them that cannot be as well or better done by free use of water. There is no doubt at least that they do better when well diluted, and possibly still better if left out entirely.

Then comes castor oil. It probably is one of the least harmful of purgatives, but it does not act by its soothing, oily properties, or mechanically. Its action is due to ricinoleic acid, and the action of this ingredient is compared to the active principle of croton oil. Castor oil is an irritant of the same class as croton oil, only milder; its frequent use, if given in overdoses, will cause enteritis.

I can hear you say, "Are we expected to believe that castor oil, that has stood the test of a century, is to be classed as a dangerous remedy?" I would not go too far, nor do I deny that cascara, rhubarb and perhaps other laxatives do have a place in our armamentarium. It would be a bold man, in view of their general use, to deny this; but I do claim that they are much over-used. This is not new at all, since warnings against the abuse of purgatives have been often repeated for many years.

I do claim, however, that, notwithstanding repeated warnings, both physicians and surgeons use purgatives in a routine, perfunctory way, very much in excess of what they should. They not only exhibit them when they do no good, but often injure their patients. Do not most patients who consult a medical man get a purgative as the first prescription? And it is a notorious fact that practically all surgeons give a cathartic as the first step toward a preparation for an operation of any kind, be it for ovarian tumor or an ingrowing toe-nail. About all the advance we have made in this line is the eliminating of the drastic drugs. I know of no way to express what the extent of this abuse of these drugs is. If I would express them in figures, a guess only, I would say that somewhere between five hundred and one thousand doses were given where one was really needed. A little startling, I admit, but, soberly, I do not think it far wrong.

I do not pretend, from my limited knowledge, to be able to give definite indications and contraindications for the exhibition of purgatives; that will require careful clinical and experimental study to do it with any accuracy. I can, however, point out a few things which cannot be done by purgatives.

The medical man is in error more frequently in the use of laxatives for chronic constipation and obstruction of the bowels, either acute or chronic. Before discussing these, I want to protest against the routine use of cathartics in acute cases, which we so often assume are due to an auto-intoxication, and that it is a poisoning from the intestinal canal, a position far from tenable. Boas says: "The importance of intestinal auto-intoxication as an etiological factor of disease is still in doubt."

These toxins are generally far out of reach of a laxative. I am well aware that I am on dangerous ground, because we know so little about auto-intoxication. This being the case, is it not better to be careful in the use of drugs whose actions we know little of, in diseases of which we know less (Holmes)? In every case ask yourself what are you to expect. Is the dose indicated? If in doubt, do not give it.

No one has seriously claimed that purgatives or laxatives cure constipation. On the contrary, practically all those best able to speak say that they do harm, and their habitual use is the most potent cause of constipation. Spastic constipation is often caused, and always aggravated, by them.

We should try to discover the cause, and we will find most of them will be due to carelessness; next, errors of diet; then a small portion to a mechanical difficulty which should be overcome surgically, and about the same proportion to what we call atony, because we do not know anything about it, and in those few our ignorance compels us to give a laxative.

If, again, I may be allowed to put my guess into figures, I would say (and this is based on the record of about three hundred cases in which this point has been noted) that 98 per cent. would come under the first and second headings; 1 per cent. under the third, and 1 per cent. under the last. In the work of a general practitioner, the proportion of the last two will, I believe, be still smaller.

In acute ileus and intussusception purgatives are potent for evil, and, worst of all, the time for successful operation is often allowed to pass while waiting for the effects of drugs. In spite of all that has been said, such neglect is still far from uncommon, as every surgeon will testify. Purgatives are relied on in chronic obstruction very often until the lesion has occluded the bowel, and the patient's condition is such that operation is hazardous before the surgeon is consulted. These chronic obstructions are often due to cancer, it is true, and little hope can be held out. Still, early surgical interference would save some of them, and, what is more important, a proportion of the malignant disease is engrafted on benign lesions, and an early removal would result in complete recovery. I recently saw a patient with cancer of the sigmoid, with metastases to an extent that radical removal was impossible, who had had distinct symptoms of partial obstruction for fifteen years, during which time he was treated with purgatives by a number of physicians, none of whom intimated that anything else could be done for his relief. It is highly improbable that this disease was malignant for so many years.

The time has come when on the general practitioner, and not on the surgeon, will rest the blame for the unfavorable results in these bad cases.

The surgeon, in spite of his boasted medical nihilism, is not behind his medical brother in the thoughtless misuse of purgative drugs. I have yet to find a hospital in which it is not the practice to give every patient who is to undergo any kind of an operation a purgative, if time will permit, and in large majority they are used afterward; the practice varying from giving a dose just before the operation and continuing immediately after with salines, until the bowels move, preferably in the first twelve to twenty-four hours (Byford) to several days after.

If we can judge by works and articles on surgery, it must be a very small percentage who escape a cathartic both before and after operation. If the operation is not on the intestinal canal, why should we give it? And if it is on the prima via, what good does it do?

It goes without saying that a large proportion of cases who come to operation have no trouble with their stomach or bowels. Most of the directions on this subject are vague, and do not rest on fact. We are told to "unload the bowels." If there is loading of the bowel, it is in the colon. The small intestine has always fluid contents, and its peristalsis is almost as constant and regular as the rhythm of the heart. Stasis in the stomach and small intestine is due, with only the rarest exceptions, to mechanical obstruction, for which drugs are useless. It took us ten years or more to get away from the practice of trying to purge out an appendiceal abscess. Fecal impaction in the colon is not common, and when it does occur, purgatives are poor remedies, and a single purge does not remove it; on the contrary, it adds fluid and more germs, which make the development of toxins more certain.

I think a moment's reflection will convince most of you that for operations other than those on the digestive tract an enema is better and more rational, whatever the condition may be. Even this is often superfluous, but patients are more comfortable to have the lower bowel empty, and we do not have to disturb them so soon afterward.

How is it about operations on the intestine? If the patient is long enough under observation to have a purgative, say twenty-four hours, his condition will be better and he will surely feel better if he is fed on a light, digestible diet, and allow his bowel to empty itself; if it does not, an enema will clear the rectum and sigmoid. If impaction of feces in the colon exists, several days at least will be necessary to remove it, and should be taken if the condition will permit.

As pointed out above, these drugs act as irritants; they increase the fluid in the alimentary canal, they increase the growth of bacteria and render them more virulent, and as a consequence increase the formation of gas. They weaken a patient, and bring him to operation at least more uncomfortable than he would have been without them. Of these reasons, the stimulant given to germ life is most important, and with it we irritate the mucous lining of the bowels, making it less resistant.

Are we not doing with the intestines exactly what we formerly did with the skin. We would scrub it, disinfect it, until normal resistance was diminished or destroyed. Our results have been better since more rational methods were adopted. Why not do the same with the alimentary canal? Let nature alone unless the evidence is clear she cannot do her part.

After the operation the rule should be the same; only give laxatives when clearly indicated. There is no good evidence that they have any power to limit infection if it has taken place; in fact, it is debatable if its possibilities for harm are not far greater.

If no complication exists, they are rarely required. The bowels will move spontaneously as soon as food in sufficient amounts is taken. In an article on the abuse of purgatives (American Journal of Obstetrics, Vol. liv, No. 6) I mentioned the fact that impaction of the rectum was troublesome, but not a serious complication, in some cases. This can usually be remedied by getting the patient out earlier, but if this does not suffice an enema will be of service.

I have operated on nearly one thousand patients for a variety of diseases, a large portion abdominal, without the "usual purge" before, and of these not over 5 per cent. had one after the operation. I am sure my results have been as good, and I think better, than when the giving of a purgative was a routine. The patients have been more comfortable, and the most marked advantage is that they have much less tympany. I cannot say the vomiting is less, but the convalescence is more comfortable, and fully as rapid as before.

In conclusion, I want to state that I do not deny that purgatives hold an important place in our armamentarium, and we could not do without them. I protest, however, against their routine use, for they are powerful for evil as well as good. I do not pretend to give final conclusions on so great a question, but think it is high time we were making a definite study of the indications and contraindications of purgative medicines. They are not curative; at best, they can only give temporary relief, and when we have to repeat them it is good evidence that there is some underlying disease we have not reached. This may be almost anything, but very frequently is an organic lesion, such as tuberculosis, cholelithiasis, nephritis, or the like. In fact, in the early stages of almost all serious diseases, patients are injured and much valuable time is lost by a course of purgatives which most of them are put through. Their routine use in medical practice is indefensible; in surgical practice, is absurd.

